# Between the Domestic and the Foreign: The KGB and Soviet Muslims in the Late USSR

**DOI:** 10.1177/00220094251322187

**Published:** 2025-03-02

**Authors:** Vassily Klimentov

**Affiliations:** 27217 Department of History, University of Zürich, Switzerland

**Keywords:** Soviet Union, Islam, foreign policy, KGB, Azerbaijan

## Abstract

This article examines the ambivalence of the Soviet authorities’ attitude to and policy toward Soviet Muslims in the 1970s and 1980s. Soviet Muslims were an asset for Soviet foreign policy in Muslim countries, serving as KGB operatives and as diplomats in the Middle East, Iran, South Asia, and Afghanistan and proved generally loyal to the Soviet state. However, some Soviet officials, notably in the KGB, did not fully trust Soviet Muslims. They kept them in junior positions abroad, suspected them of foreign sympathies, and continued to monitor their activities at home. This dichotomy was incarnated in Soviet Muslim border regions such as Azerbaijan. Azerbaijanis were key to Soviet intelligence operations in Iran, but the KGB suspected them of sympathies for the Iranian Islamic Revolution. Some Soviet Muslims, including those in Azerbaijan, did root their opposition to the Soviet state in Islam.

The study of Islam in the Soviet Union has experienced a revival over the last 20 years. The opening of the archives of the Soviet agencies overseeing religion, including the Council for the Affairs of Religious Cults and its successor the Council for Religious Affairs (CRA), and the Spiritual Administration of the Muslims of Central Asia and Kazakhstan (SADUM) that oversaw Islam in Central Asia, research among post-Soviet Muslims and engagement with new literary sources have produced a new narrative which has shown how Islam and communism often co-existed in Soviet Muslim regions.

This article continues the reassessment of the Soviet relationship with Islam by showing how the Committee for State Security (KGB) and the Ministry of Foreign Affairs (MID), two institutions between which there was overlap because many diplomats were KGB agents, mobilized Soviet Muslims.^1^ It shows how many Soviet Muslims engaged with the state, reconciling their ‘Muslimness’ with the imperatives of service. Many Soviet Muslims collaborated with the KGB and MID as envoys in Muslim countries and often later at home as Islamic scholars, part of what the Soviets called the Muslim ‘clergy’. In that sense, many Soviet Muslims had seemingly no conflicting loyalties despite the official opposition to religion in the Soviet state. Conversely, in many instances, Soviet authorities, including the KGB, trusted Soviet Muslims as much as non-Muslims to advance Soviet interests.

This article, nevertheless, nuances the story of the convergence between Islam and communism by highlighting that not all Soviet Muslims were eager to cooperate with the Soviet authorities. Some saw issues with advancing the interests of an atheist state in Muslim countries or collaborating with institutions at home which proclaimed that their ultimate goal remained the disappearance of religion. Even those Soviet Muslims who did cooperate with the state undermined it at times, willingly or not. The article also shows that the Soviet authorities’ reliance on Soviet Muslims had limits because the USSR remained an atheist state. The KGB continued to monitor and repress the more spiritually-minded Muslims in the Soviet Union. Suspicions, likewise, lingered toward Soviet Muslims who acted as agents abroad. Ultimately, Soviet authorities feared, at times with good reason, the effect of the Islamists’ rise in Iran and Afghanistan on Soviet Muslims.

As it tells its story of convergence and tension between Islam and communism in the USSR, the article emphasizes one central theme: that for Soviet authorities, Muslims were a matter of both domestic and foreign policy. Soviet Muslims were sometimes perceived as an opportunity in foreign policy and sometimes as a threat in domestic policy. This foreign-domestic tension was especially visible in Soviet borderland Muslim regions such as Azerbaijan whose citizens were key to Soviet intelligence operations in Iran but suspected by the KGB of sympathies for the Iranian Islamic Revolution.

To support its argument, the article relies on the Mitrokhin Archive. These are KGB documents collected by Vasily Mitrokhin, a KGB archivist who defected to the United Kingdom in 1992, and held in Cambridge and the National Archives in London. The Mitrokhin Archive, most famously used in a monograph by Mitrokhin and Cristopher Andrew, remains a source historians must treat with caution.^2^ Mitrokhin's documents are transcriptions and summaries of files from closed KGB archives. While some of his information has been cross-checked with the opening of new archives, Mitrokhin seems at times ‘to overstate the subversive nature of Soviet policies and KGB activities’.^3^ Considering this, this article uses Mitrokhin's documents to only provide a general picture of the KGB's operations in Muslim countries and attitude toward Soviet Muslims. In addition, the article draws on Soviet archives in Moscow, memoirs by Soviet officials, and other sources.

The article is organized into five sections. It starts with discussing the transformation of the history of Soviet Islam. It afterward examines the role of Soviet Muslim agents in the Middle East and the connections between domestic and foreign policy. It then moves to analysing the role of Soviet Muslim agents in the KGB and MID in Iran, in South Asia and in Afghanistan. In its fifth section, the article shows how the KGB remained concerned about Islamic contagion streaming to the USSR from Iran and the protest potential of Soviet Muslims.

In recent years, historians have rethought the relationship between communism and Islam among Soviet Muslims. Challenging the presumed opposition between the two ideologies, this interpretation shows how Soviet Muslims reconciled the practice of Islam in the private and public spheres with adherence to the tenets of communism.^4^ It deconstructs the dichotomies inherited from Cold War-era academia which used to govern the study of Soviet Islam. Eren Tasar demonstrates the limitations of the urban/rural divide in analysing Soviet Islam and the mistake of seeing all urban Muslims as secularized and only integrating Islam culturally.^5^ Conceptualizing Soviet Islam as a ‘degraded’ religion that had to ‘adapt to Soviet conditions’ and become a cultural attribute for urban elites fails to account for the diversity of Soviet Muslims’ experiences.^6^ As Paolo Sartori explains, the nature of the Soviet and Muslim dyad was never clear throughout Soviet history.^7^

Although not all Soviet Muslims were ‘engaged in perfecting themselves … in religion or even thinking consciously about the sacred’, religious-minded people were an integral part of the population of the USSR.^8^ In parallel, some Soviet Muslims ‘only kept in reserve stories, practices and prescriptions derived from an Islamic episteme, which they could deploy in times of need’.^9^ These Soviet Muslims ‘did not look to Islam for moral guidance’ but used it as a form of ‘cultural capital to advance their careers in the Soviet Union’.^10^ The diversity of Soviet Muslims makes it difficult to ascribe collective attitudes and perceptions to them as a group but it stresses how they were agents in the Soviet system and influenced it in various ways. It was possible, even common, to be both Soviet *and* Muslim in the USSR.

Historians nuance the dichotomy postulated by Cold War scholars between what Soviet officials called the ‘registered’ Muslim ‘clergy’ who were controlled by Soviet institutions dealing with Islam and the ‘unregistered’ who operated illegally. The boundaries between the two groups were permeable and some Soviet institutions also cared about the interests of the ‘unregistered clergy’. This led to a reassessment of the protest potential of the non-registered Islamic scholars, the majority of them did not oppose the Soviet system, paid their taxes and maintained relations with official institutions such as SADUM.^11^ Muhammadjan Hindustani, who led Islamist teaching circles in Uzbekistan from the 1970s, argued that ‘under Soviet rule, Islam in Central Asia had remained intact, for Muslims could cultivate their faith, perform rituals, and transmit their own culture’.^12^ Attitudes such as his added to the diversity of Muslim experiences under Soviet rule. Ultimately, Islam's vitality and the large number of unregistered places of worship in Central Asia participated in forcing the Soviet authorities, notably through the intermediation of SADUM, to compromise on anti-religious policy, including by registering more mosques in the 1980s.^13^

Two other factors helped create the conditions for Islam's vitality in the post-Second World War Soviet Union. First, while the Soviet authorities used religion ‘as a category for understanding the population’ at home, Islam also mattered in foreign policy.^14^ Starting with Nikita Khrushchev, who led the Soviet Union between 1953 and 1964, Soviet Muslims played a role as Moscow built relationships with Muslim countries. SADUM and similar institutions in the Caucasus and Russia developed an outreach strategy toward foreign Islamic institutions, including in countries with which the USSR had little official relationship such as Saudi Arabia.^15^ Presented as ‘cultural Muslims’, Soviet Muslims embodied the successes of Soviet modernization and testified to how it benefitted the entire population of the Soviet Union. The Soviets also displayed their own Muslims to win over Muslim officials visiting the Soviet Union, including especially from countries where Islam was the official religion. This strategy prevailed until the end of the Cold War. During the Soviet-Afghan War (1979–89), the Soviet Union dispatched hundreds of Muslim advisers to Afghanistan where they became ambassadors for the Soviet modernization project. In parallel, despite the backlash from the Afghan conflict, Moscow worked to expand its influence in the Muslim world in the 1980s.^16^

Second, Soviet policy on religion at home was marked by both inconsistency between the centre and the periphery and, as a consequence, leniency. After the Khrushchev era, despite occasional intensifications of atheist propaganda like following Mikhail Gorbachev's rise to power in 1985, the Kremlin did not call for the violent repression of practising Muslims. This increased tolerance was upheld by local authorities in Muslim republics. Originating among local elites, they reported to Moscow that de-Islamization was progressing while themselves engaging in Islamic rituals. As Sartori explains, ‘CARC commissioners and [Communist] Party cadres at all levels turned a blind eye at the fact that unregistered mosque communities […] submitted reiterated applications for registration […] that underground Islamic schools recruited hundreds of students; that Islamic literature circulated’.^17^ The Soviet-Afghan War also testified to the accommodation between communism and Islam and to the Kremlin's disregard for religion. Few protests against the war occurred in Soviet Muslim regions and few Soviet Muslim soldiers deserted to the Mujahideen in Afghanistan.^18^

While it has provided invaluable insights and helped rethink the relationship between Islam and communism in the Soviet Union, the historical narrative described above is not without its blind spots. One criticism it faces is that it has pushed the argument about the convergence between Islam and communism too far. It is as if, after years of historians arguing that there was a complete opposition between communism and Islam, the pendulum had now swung too far to the other extreme. Although both Tasar and Sartori recognize that there were communist party hardliners pushing atheism in Soviet Muslim regions in the 1970s and 1980s, they tend to underplay their importance.^19^

There is, however, evidence of the strength of anti-Islamic policies and attitudes in the Soviet Union even in the 1980s, of the Soviet authorities’ – especially the KGB – lingering suspicions toward Soviet Muslims, of Soviet Muslims complaining that they had lost much of Islam under Soviet domination, and of urban elites relating to Islam only as a cultural factor. In his historical study on Central Asia and his criticism of Tasar, Adeeb Khalid articulates these points most clearly.^20^ Although he insists on the strength of Islam in Central Asia, Stéphane Dudoignon likewise shows that there were overlooked forms of dissidence among Soviet Muslims based on a study of gnostic poetry among Central Asian Sufis.^21^ Clearly, there was discontent among Soviet Muslims concerning Soviet policies on Islam.

There is also evidence that policymakers in Moscow strongly opposed Islam. In his memoirs, Anatoly Chernyaev, Gorbachev's foreign policy adviser who himself held biased opinions toward Soviet Muslims, gives examples of Politburo members’ atheism, of the orientalist stereotypes they associated with Soviet Muslims and of their lack of knowledge of the situation with Islam in Soviet Muslims regions in the 1980s.^22^ To Andrei Gromyko, the long-serving Soviet Minister of Foreign Affairs, Islam was the religion that ‘created the most fanatics’.^23^

Other authors provide evidence supporting these arguments based on Soviet Muslims’ experiences during the Soviet-Afghan War. Timothy Nunan highlights how some Tajik advisers deployed to Afghanistan felt that they were inadequate Muslims compared to the Afghans.^24^ Vassily Klimentov demonstrates how, unlike the authorities in Moscow, KGB and military officers and Central Asian communist elites such as Uzbekistan's Islam Karimov had concerns about an Islamist threat engulfing Central Asia after 1988. He also stresses that many Soviet Muslims deployed as advisers to Afghanistan had only superficial knowledge about religion and that Soviet officials had no trust in Soviet Islamic scholars, even though those visited Afghanistan.^25^ Similarly, regarding the North Caucasus, although the region is understudied compared to Central Asia, Chernyaev explains how Islam drove opposition to serving in the Soviet forces in Afghanistan.^26^

Soviet Muslims played a role in Soviet overt and covert policy in Muslim countries as part of MID and official delegations and clandestine KGB activities. Regarding covert action, a central function of the KGB abroad was to conduct so-called ‘active measures’ (AMs) that included espionage, the recruitment of agents, disinformation, propaganda, sabotage, support to armed groups and other covert activities.^27^ It particularly targeted Muslim countries that had become a prime battlefield of the Cold War. Moscow wished to consolidate its influence in the Middle East and South Asia and undermine that of the United States which had surged following Egypt's break with the USSR in the mid-1970s. Enhancing the Soviets’ role in the Middle East became even more important following the intervention in Afghanistan which saw most of the Muslim world led by Saudi Arabia, Pakistan and Egypt condemn the Soviets and support the Mujahideen against the Afghan communists.

The KGB's operations involving Soviet Muslims had a distinctly hybrid character linking domestic and foreign policy. Handpicking with the CRA Muslims for Hajj delegations and pilgrimages to Iraq, the KGB used Soviet Muslims to gather information, improve the image of the USSR and recruit and infiltrate operatives in Muslim countries. Hajj delegations like the one in 1966 would visit other Muslim countries on their way back from Saudi Arabia, disseminating positive information about Soviet Muslims. By 1970, some 60 KGB operatives had travelled on Hajj delegations.^28^ After their return to the USSR, Hajjis used their newfound status to strengthen the legitimacy of Soviet institutions overseeing Islam and advance Soviet policies on religion. The dealings of the pilgrims with the KGB did not mean, however, that some of them were not spiritually minded Muslims who had to compromise for a chance to conduct the Hajj.^29^ This ambiguity defined the situation of many Soviet Muslims. Meanwhile, the Hajj showed how Soviet intelligence agencies trusted Soviet Muslims.

Beyond this, Muslims from Uzbekistan and other republics attended Islamic schools in Egypt, with a preference for the famous Al-Azhar institution, Saudi Arabia, and pro-Soviet Syria and Libya. The CRA and KGB instructed these recruits to work with foreign Islamic leaders and organizations such as the Saudi-led Muslim World League to present a more positive image of the Soviet Union. They also had to train in Islam to become respected Islamic authorities at home, part of the ‘registered clergy’ that liaised with the KGB. Such Islamic scholars who were members of SADUM and similar institutions would be central in the reception of foreign Islamic officials visiting the Soviet Union.^30^ The organization of these visits inevitably built on the contacts established by Soviet Islamic envoys abroad. Also, the KGB instructed Soviet Muslims sent to Muslim countries to bring a selection of Islamic literature to the Soviet Union to refine the training of new Soviet Muslim agents.^31^ The system therefore perpetuated itself as returnees trained aspiring agents. In fact, the main limit to Soviet Muslims’ involvement with the KGB was that they did not rise to the position of station chief in Middle Eastern countries or to senior positions on the Middle East in the KGB's office in Moscow.^32^

In addition to working with the KGB, Soviet Muslims were involved in diplomatic roles in the Middle East, including at times as ambassadors. Nuritdin Mukhitdinov served as ambassador to Syria from 1968 to 1977, an unusually long stint for a Soviet diplomat. A former head of the communist party in Uzbekistan, he played on his Muslim heritage to gain credit in Syria. According to his British counterpart, Mukhitdinov ‘knew a few words of Arabic and always dragged them out rather ponderously to greet [his diplomatic colleagues]’.^33^

Soviet Muslims also worked in other Middle Eastern countries during the Cold War. Although many held positions in Soviet embassies, their number as ambassadors was small enough for them to be easily listed. The Uzbek writer Sarvar Azimov was Ambassador to Lebanon between 1969 and 1974. Rafik Nishanov was Ambassador to Jordan between 1978 and 1985 before heading the communist party in Uzbekistan. Mirzo Rakhmatov, a leading member of the communist party of Tajikistan, served as ambassador to the Yemen Arab Republic between 1966 and 1972. Avdy Kuliev, a career Soviet diplomat from Turkmenistan, was the chargé d’affaires in Oman and Qatar between 1988 and 1989 as the USSR improved relations with pro-Western Muslim countries during Gorbachev's era. As clear from the list, most ambassadors had made it to such positions after having proved themselves as reliable party apparatchiks at home. Other Soviet ambassadors in Middle Eastern countries from 1945 to 1991 were, however, mostly Slavs.

Likewise, Soviet Muslims were often part of delegations from Moscow to Muslim countries but rarely led such delegations, a consequence of their limited presence in the higher echelons of Soviet power.^34^ In Joseph Stalin's times, Mir Jafar Baghirov, who led the repressive regime in Azerbaijan, had been the only Muslim to be a Candidate Member of the Politburo in 1953. Until the final years of Gorbachev's *perestroika*, only four more Muslims joined the Politburo: Mukhitdinov between 1957 and 1961; Dinmukhamed Kunaev, the head of the party in Kazakhstan, between 1966 and 1987; Heydar Aliyev, the head of the party in Azerbaijan, between 1976 and 1982 as Candidate Member, and then as Member until 1987; and Sharof Rashidov, the head of the party in Uzbekistan, as Candidate Member between 1961 and 1983. These four Muslim leaders held influence within the Soviet system but it is striking how few Soviet Muslims made it to the top. Their influence also had limits. Kunaev, Aliyev, and Rashidov were, for example, marginalized in the discussions that led to the Soviet intervention in Afghanistan.^35^

In the 1980s, the Soviets, nonetheless, played on Aliyev's Muslim background when sending high-level delegations to Muslim countries. As noted by British diplomats in Damascus when Aliyev came to ‘troubleshoot’ the Soviet-Syrian relation in 1984, his ‘Azerbaijani nationality (and hence Muslim background) may [have helped] implicitly to make a point about Soviet proximity to and common interests with the Middle East’.^36^ Conversely, when leaders from Muslim countries visited the USSR, they were treated to meetings with the few senior Muslim officials in Moscow outside the Politburo. In 1978, a visiting Jordanian prince met Sabir Niyazbekov, the Kazakh Deputy Chairman of the Praesidium of the Supreme Soviet.^37^

Few Soviet Muslims worked in top Middle East policymaking roles in Moscow. As clear from British-Soviet consultations since the late 1970s, no senior Soviet official dealing with Muslim countries in the MID was a Muslim. Likewise, few of the journalists covering the Middle East in *Pravda* and in other major newspapers or academia were Muslims. British diplomats who came to meet the experts of the Moscow-based Oriental Studies Institute, which was closely linked with diplomatic and intelligence circles, did not encounter any Muslims.^38^

Given many Soviet Muslims’ interest in Islam and Muslim countries, the knowledge that some Central Asian Muslims had of Arabic and Persian and the role of Soviet Islamic scholars in reaching Islamic institutions abroad, one could have expected to find Soviet Muslims working on the Middle East in leading roles in the MID, Oriental Studies Institute and similar institutions in Moscow. The fact that this was not the case underscores the Soviet authorities’ ambivalent attitude towards Soviet Muslims and suggests that they could have better exploited the potential of Soviet Muslims to advance Soviet foreign policy abroad.

Considerable Soviet undercover activity focused on Iran, which Moscow saw as the pivotal country for US influence in the Middle East. During the 1970s, the KGB ran espionage activities and set up elaborate plots to undermine the Iranian-American alliance. According to Yuri Andropov, the KGB's chief who would head the USSR after 1982, it did not matter that Soviet-US relations had improved. Détente had opened up even more opportunities for operations against American interests.

The Soviets then worked to discredit pro-US officials among the Iranian political and military elite. AMs included disinformation in Iranian media and the recruitment of KGB operatives among the local population for surveillance and sabotage activities. A much-celebrated operation had the KGB forge a letter attributed to the US embassy that was then leaked to the Iranian authorities to undermine trust between Washington and Tehran.^39^ The KGB measures met with varying success in the mid-1970s leading to the recruitment of senior operatives such as a General in the Iranian general staff, and, on the other, to setbacks that saw some Soviet agents, including that General, being arrested and executed by the Shah's secret police.^40^ According to British diplomats, the Shah saw Moscow's hand behind every disturbance by 1976, fearing a Soviet attack on Iran. In fact, while Soviet AMs had increased in Iran, the KGB's reach in the 1970s remained limited according to Vladimir Kuzichkin, a KGB operative in Tehran who would defect to the West.^41^

Taken aback by the Islamic Revolution in 1978–79, the Soviets tried to take advantage of it to turn Iran against the US.^42^ Supporting its communist allies from the People's Party of Iran (Hezb-e Tude-ye Iran, known as Tudeh), Moscow worked to not antagonize Iran's new religious authorities. Andropov instructed operatives to be careful to not endanger bilateral relations while Moscow hoped to build an anti-American front with Tehran.^43^ Following the start of the Iran-Iraq War in September 1980, Moscow even risked its relations with its Arab allies and Baghdad in an attempt to stay neutral in a conflict where traditional Cold War alliances were blurred. As the FCO observed, the Soviets had no doubt ‘calculate[d] that Iran [was] a better prize than Iraq, which had in any case proved an unreliable ally’.^44^

In the early 1980s, the self-imposed or forced exile of some Soviet agents from Iran and the repressions that struck others, notably in the Tudeh, complicated the KGB's AMs. As the Soviets became bogged down in Afghanistan, the KGB worked to limit Iranian support to the Mujahideen by keeping Iran occupied with other affairs and spreading disinformation about a Pakistani-US-Mujahideen alliance directed against Iran. The KGB ran some of its activities in Iran using the Palestinian Liberation Organization (PLO) and its Chairman Yasser Arafat.^45^ The use of the PLO further suggests that the Soviets could not solely rely on their depleted pool of agents.

Even more than in Arab countries, KGB operations in Iran testified to the connection between domestic and foreign policy. Despite its heads in Tehran being traditionally Slavs, the KGB had relied on Azerbaijanis and other Soviet Muslims in Iran since before the 1970s.^46^ Some of these operatives, notably from among the Azerbaijanis who were predominantly Shia Muslims, had been infiltrated as Shia pilgrims. By the 1980s, more Soviet Muslims were sent to Iran.^47^ Tehran, in return, complained about Soviet attempts to destabilize and run disinformation campaigns in Iran from Azerbaijan. High-level Afghan officials fleeing after the Soviet intervention similarly claimed that Soviet spies were numerous in Iran, including in the inner circle of Imam Ruhollah Khomeini, Iran's new leader. Other sources confirmed that the Soviet Union used Azerbaijanis, Turkmens and pro-Soviet Afghans to infiltrate Iran, be it as Tudeh sympathizers or members of the Shia clergy.^48^

In his memoirs, Kuzichkin confirms the KGB's reliance on Soviet Muslims in Tehran, including on operatives from Azerbaijan. He notably recounts the story of agent ‘Vagif’, a man born in Baku in a family of Iranian emigrants whom the KGB infiltrated into Iran. Remarkably, before sending him to Iran, the KGB dispatched ‘Vagif’ to Kuwait. Mingling with the Iranian diaspora there, ‘Vagif’ made valuable contacts and strengthened his KGB cover which explained his long absence from his home country. The KGB then transferred ‘Vagif’ from the Soviet Union to Iran using the Soviet passenger ferry cruising on the Caspian Sea whose captain was also an Azerbaijani KGB agent.^49^

Other Soviet Muslims also worked at the embassy in Tehran in the late 1970s and early 1980s. Among senior officials, the representative of the Central Committee of the CPSU in Tehran was from Kazakhstan and, to the shock of Kuzichkin and his colleagues, had relatives in Iran whose existence he had hidden from Soviet authorities. Likewise, the representative of Sovexportfilm, the Soviet film production and distribution company, was Azerbaijani.^50^ In addition to Soviet Muslims, an Armenian representative worked at the embassy, facilitating the repatriation of Iranian Armenians to the Soviet Union. The KGB, meanwhile, kept an eye on all Iranians who may have had relatives in the Soviet Union.^51^

By the 1980s, Azerbaijan was thus consolidated as a hub from where Soviet agents could be recruited and propaganda disseminated to Iran and where Tudeh operatives could be trained.^52^ After Iranian Islamist authorities decided to eliminate the Tudeh, the Soviets tried to smuggle its leaders to the USSR over border crossings in Azerbaijan and Turkmenistan. Uzbekistan and Tajikistan also provided shelter to Iranian leftists in the 1980s.^53^

Overall, the KGB's reliance on Azerbaijanis and other Soviet Muslims to advance Soviet interests in Iran testified to the trust Soviet authorities placed in Soviet Muslims. It showed how most Soviet Muslims had no divided loyalty, cooperating with Soviet intelligence agencies against their co-religionaries or, at times in Iran, groups with whom they shared the same ethnicity. Yet, the same limits as for the rest of the Middle East are apparent in Soviet Muslims’ engagement in Iran. KGB station chiefs and MID ambassadors in Iran were ethnic Slavs. Beyond this, there were signs that some Slav operatives, such as Kuzichkin, saw their Muslim colleagues through the prism of orientalist stereotypes.^54^

Relying on Muslim operatives and diplomats, the USSR played up its respect for Islam to improve its image in Iran. Yet, because of its ties with the Tudeh and state atheism, this strategy had limits. As the FCO reported, ‘Soviet attempts to play the Muslim card have cut no ice with the Iranian Government’.^55^ The Soviet policy in Iran was further complicated by diverging assessments in Moscow of the compatibility between communism and the Iranian Islamist leadership. The latter contributed to contradictory statements from Soviet officials, notably Soviet Muslims who, at times, had an agenda distinct from the Soviet leadership. In a speech to foreign ambassadors in 1982, Aliyev, surely playing to his local constituency, voiced his sympathy for the idea of a ‘great Azerbaijan’ that would encompass Iranian territories.^56^ The awkward comment had to be quickly withdrawn afterward but its impact lingered, especially as Iranians and foreigners suspected the Soviet Union of planning to use separatism to advance its interests in Iran.

Because it was the Mujahideen's main supporter and hosted most Afghan refugees, Pakistan was a prime target of KGB AMs in the 1980s. These built on operations that existed in the 1970s but were done on a smaller scale given Pakistan's lesser strategic importance for Moscow compared to Iran. The KGB then ran disinformation campaigns targeting Islamic leaders seen as anti-Soviet such as Abul A'la Maududi. This interestingly showed the KGB's concern with early Islamist thinkers besides Iran. Moreover, the KGB's disinformation and intelligence activities aimed at scaring the Pakistanis with another partition after the secession of Bangladesh in 1971. As Soviet operatives hinted, the United States and Iran's Shah would welcome such a weakening of Pakistan. Like in Iran, the Soviets tried to discredit pro-Western elites.^57^

The AMs in Pakistan were intensified following the intervention in Afghanistan, even though the loss of local agents rendered them more complicated. As the KGB lamented, Pashtuns and Baluchs who used to see the USSR and Afghanistan as natural allies in their quest for independence now turned against them.^58^ Still, the KGB designed a strategy to limit Pakistani support to the Mujahideen. The latter included a campaign of publications aimed at highlighting that the United States was instrumentalizing the Soviet-Afghan War to divert attention from other issues. It also attempted to scare the Pakistani elites and Muhammad Zia ul-Haq, Pakistan's authoritarian leader, with either an Iranian-type Islamic Revolution, troubles fomented by the Mujahideen, or another conflict with India.^59^ In parallel, the Soviets tried, with limited success, to undermine the emerging pro-Mujahideen international front led by Pakistan. More ominously, the Soviets used covert channels to threaten the Pakistanis with either airstrikes or with arming independentist movements inside Pakistan if Islamabad became too supportive of the Mujahideen.^60^

The KGB also targeted Bangladesh with its disinformation. Articles planted in the local press argued that the US was instrumentalizing the Soviet-Afghan War to detract attention from the problems in Bangladesh and from the Iranian Islamic Revolution, raised the spectre of another conflict with Pakistan, and minimized the Soviet presence in Afghanistan. As in Iran, the KGB used elements of the PLO to disseminate supportive statements about the Soviet Union in South Asia.^61^

Beyond this, despite it being a Soviet ally, KGB's AMs targeted India and, in particular, its Muslim minority. The KGB was willing to incite a new armed confrontation between New Delhi and Islamabad to detract Pakistan from Afghan affairs and bring an alignment between New Delhi and Kabul. In addition, KGB operatives placed articles in the local press to prevent an improvement of Indian-Chinese relations and to boost Indian support for Afghanistan.^62^

In its operations in South Asia, the USSR also mobilized Muslim agents. This strategy was, however, limited by the fact that Soviet Muslims did not share cultural and ethnic links with local populations in Pakistan, Bangladesh and India. Still, Uzbeks were sent to India to pose as local Muslims, even though they were not viewed as ‘real Muslims’ as the Mitrokhin Archives explain.^63^ This blunt statement testified to the Soviet thinking about Soviet Muslims. The KGB trusted them to some extent because it saw them as ‘cultural Muslims’ and so there was limited risk of Islamic contagion from operating in Muslim countries.

As in the Middle East and Iran, Soviet Muslims in South Asia were underrepresented in top positions in the MID and KGB. Azimov, who became the ambassador to Pakistan in 1974, was the only Soviet Muslim to serve as an ambassador to the region during the Cold War and the Uzbek Gumer Agzhigitov who was posted in India – not a majority Muslim country – in the 1950s was apparently the only Soviet Muslim to be a KGB station chief.

During the Soviet-Afghan War, KGB operations were considerable and multi-faceted, dwarfing in scope the KGB's activities in other Muslim countries. Discussing them in detail lies beyond the scope of this paper but Muslims clearly had a key role in intelligence gathering and contacts with the population, representing the sort of modern ‘cultural Muslims’ that the USSR wanted Afghans to emulate. Many Tajiks and Uzbeks who spoke Persian, one of the languages of Afghanistan, thus became translators, including often with the KGB.^64^ This was even the case of some unregistered Islamic scholars, spiritually-minded Soviet Muslims, who accepted to fight against their co-religionaries despite their reluctance to do so.^65^

Various studies on the Soviet-Afghan War also state that Soviet Muslims, even if they had reservations about the conflict, served loyally in the Soviet forces.^66^ Likewise, Soviet Islamic scholars did not shun making trips to Afghanistan to voice the official line of the party in speeches to the Afghans. Usmonjon Rahimjonov, a prominent Uzbek Islamic scholar from SADUM who had studied at Al-Azhar, came to Afghanistan in 1984 to tell Afghans about how the Soviets were ‘helping [them] to rebuild and develop their economy’ and enjoined them ‘not to fall under the power and domination of the imperialist countries’.^67^ While it is impossible to know about Rahimjonov's exact affiliations, one can assume that he had dealings with the KGB in the USSR, again evidencing the connections between Soviet domestic and foreign policy toward Muslims.

Other examples testify to this hybridity between domestic and foreign policy and connections across different countries in foreign policy in the KGB AMs. KGB's operations in Afghanistan thus involved the recruitment of agents to be sent to Iran, likely after training in Central Asia.^68^ Likewise, the Mitrokhin Archives provide an interesting example of how the KGB in Dushanbe took the initiative of creating a programme to infiltrate Tajik operatives as itinerant mullahs into Soviet-controlled Afghanistan. The goal was for them to acquire a credible background as Islamic scholars before the KGB could deploy them to Xinjiang to foster the Uighur Muslims’ resistance to Chinese authorities. This bold operation was cancelled after the Afghan intelligence services explained to their Soviet colleagues that itinerant mullahs did not have much prestige in Afghanistan.^69^ Still, the fact that such AMs were developed shows how the KGB brainstormed using Islam to advance Soviet interests by relying on Soviet Muslims. It also shows how central Soviet Muslims were to some of its initiatives.

As in other countries, despite their massive presence on the ground, few Soviet Muslims occupied senior positions in Afghanistan. According to Vladimir Plastun, a senior Soviet civilian adviser in the military and a Soviet orientalist who trained KGB and MID personnel, many Slavs in Afghanistan saw Soviet Muslims as not ‘sincere communists’.^70^ This did not mean that they doubted their loyalty, but rather that they saw them as not as ideologically committed to communism. Such perceptions translated into the roles held by Soviet Muslims. For instance, over the ten years of Soviet presence in Afghanistan, only two to three dozen advisers out of the 300 communist party advisers sent by Moscow were Soviet Muslims.^71^ This low proportion is especially significant as party advisers were a privileged corps that held particular sway with the Afghan authorities. Likewise, KGB station chiefs in Kabul were not Soviet Muslims, although many Central Asians served in intelligence roles.

Fikryat Tabeev, the influential Soviet ambassador to Kabul, represented a rare exception. An ambiguous figure, Tabeev arrived on the eve of the Soviet intervention. While his Muslim background likely played a role in favouring his assignment, he was first a committed ideologue who had become well-regarded in Moscow for steering the communist party in his native Tatarstan republic. As his interactions with Afghans demonstrated, he epitomized the idea of a ‘cultural Muslim’, being at times more ideological in his Marxism-Leninism than his Slav colleagues. Tabeev's profile was therefore similar to those of some Soviet Muslim ambassadors in the Middle East: they were party apparatchiks first. This, however, did not prevent them from playing on their Muslim heritage. As Tabeev would explain to Afghans, he was well-versed in Islamic traditions because he was a Muslim himself.^72^

The KGB had longstanding fears of foreign forces instrumentalizing Islam in Central Asia, the North Caucasus and Azerbaijan against the authorities. The Islamic Revolution in Iran made these apprehensions worse and they were exacerbated by US attempts to use the ‘Islamic factor’ to destabilize the USSR by smuggling nationalist propaganda and Qurans from Pakistan, increasing anti-Soviet Islamic radio propaganda from Saudi Arabia and, by the late 1980s, tolerating Mujahideen incursions into Central Asia.^73^ To Vladimir Kryuchkov, head of the KGB's First Chief Directorate, there were ‘religious fanatics’ among Soviet Muslims who tried to challenge the Soviet order.^74^ Ultimately, even though Islamism did not crystalize as a force of contestation against the authorities before the 1990s, its threat loomed in the minds of Soviet intelligence agencies.^75^

Soviet authorities saw Azerbaijan and Tajikistan as regions most at risk of Islamic contagion. Monitoring Soviet Muslims’ attitudes toward Iran, the FCO noted sympathies in Azerbaijan toward the Iranian Islamists. Soviet officials in Muslim regions such as Muhammetnazar Gapurow, the head of the party in Turkmenistan, started denouncing Islam more forcefully. Soviet leaders in Moscow discussed how to counter the Iranian propaganda directed at Soviet Muslims.^76^ The FCO noted ongoing Iranian propaganda across the border in 1985, 1986 and 1987. The Soviets, it then argued, ‘have, on occasion, shown considerable sensitivity to religious broadcasts (radio and television) from Iran’.^77^ By 1989, Alexander Bessmertnykh, the future Minister of Foreign Affairs, noted his ‘worries about the future of the Islamic republics in the Soviet Union’ when explaining the reasons for the USSR's rapprochement with Tehran. To him, this was the main reason for the improved ties; getting support in post-Soviet Afghanistan came only fourth.^78^ The tension with Iran over Azerbaijan remained until the USSR's collapse. Yuli Vorontsov, the influential Deputy Minister of Foreign Affairs, again blamed the Iranians for fostering trouble in Azerbaijan. ‘Some in Iran were exploiting the situation in the Caucasus, portraying it as a struggle by Shiite Moslems against Armenian Christians and also the Russians’ and nationalists were dreaming about a ‘great Azerbaijan’ encompassing territories in Iran and the Soviet Union, he claimed.^79^

The Mitrokhin files similarly show how the KGB was preoccupied with, and therefore allocated considerable resources to, monitoring the activities of Soviet Muslims. The KGB was disgruntled that atheism and the Soviet ‘progressive order’ were failing to gain traction in Soviet Muslim regions. Praise for Islam – even in an oblique manner – showed up in the press in Muslim regions. Soviet citizens visiting Muslim countries abroad, even probably as part of KGB and CRA-managed Hajj and official delegations, betrayed their religiousness. They went to mosques to pray, tried to learn more about Islam and smuggled religious books home.^80^ On their return to Central Asia, crowds of people gathered around Hajjis, leading to spikes in religiousness even among those Soviets who had not usually prayed regularly at the mosque.^81^ Activities aimed at advancing Soviet foreign policy and improving control over Islam at home thus ended up fostering increased Islamic religiosity in Soviet Muslim regions and making the Soviet authorities concerned. The Hajj was disappointing at another level for the KGB: it often did not result in the recruitment of agents abroad, including among Turkestani emigrants from the USSR in Saudi Arabia.^82^ This may suggest that the Soviet Muslims who participated in the Hajj did not fully espouse the KGB's agenda.

Beyond this, not all local reactions to Soviet Hajjs were positive. As Dudoignon notes, some unregistered Islamic scholars denounced the Hajj, ‘which was reserved in the USSR for happy-few *muftiyyat* [sic] staff and hand-picked notables’, as a ‘diversion from Islam’. Instead, they insisted on ‘indigenousness’ in Islam and disengagement from Soviet Islamic institutions.^83^ This interestingly showed how too much enthusiasm for and opposition to Soviet initiatives in Muslim countries were both an issue for the Soviet authorities. These unregistered Islamic scholars meanwhile resented the continuing atheist propaganda and restrictions on Islam at home. Their opposition passed through covert channels, including the illegal distribution of Islamic literature and unpublished Sufi gnostic poetry.

The KGB in turn responded to what it perceived as anti-Soviet attitudes. The Mitrokhin files report that the Islamic revival in Central Asia in the 1970s led the KGB to set up special ‘patriotic groups’ of agents tasked with monitoring Islam. In 1980, the KGB adopted a special directive, pushed by Andropov, to run more ‘special operations’ to deal with Islam in response to the new situation in Iran and to US propaganda.^84^ Such measures were probably reinforced after the Politburo passed resolutions aimed at reinvigorating the fight against Islam in 1980 and 1981.^85^ By 1982, as reported by Vadim Kirpichenko, the First Deputy Head of the KGB's First Chief Directorate, Andropov was personally studying a translation of the Quran to better grasp the ‘Islamic factor’.^86^

Following these edicts, the KGB intensified its measures against unregistered scholars in Central Asia. It tightened control over Islamic education abroad and in the Soviet Union, more closely monitored Hajj delegations, infiltrated additional agents among Islamic scholars in Tajikistan and other Muslim regions, and ran new recruitment campaigns in Soviet Islamic education institutions.^87^ In the 1980s, Soviet authorities jailed Islamic scholars disseminating Islamic literature in Uzbekistan as they fought against what they started calling ‘Wahhabism’. In 1986, the KGB jailed Sayid Abdulloh Nuri – an early Islamist and the future leader of the Islamic Renaissance Party of Tajikistan. Testifying to its pervasive belief that a foreign force was behind Soviet Islamists, the KGB suspected Nuri and other Tajik Islamists of working for Iran despite them being Sunni Muslims.^88^

Protests against the authorities in some Soviet Muslim republics aggravated the KGB's concerns about the alliance between Islam and nationalism, influencing the strengthening of atheism campaigns in the mid-1980s. In Kazakhstan and Uzbekistan, Soviet policymakers, including Gorbachev, blamed nationalism and Islamic fundamentalism for the disturbances.^89^ Religion, seen as predominantly a cultural factor, was strongly associated with national protests and the KGB saw the pan-Islamic threat in conjunction with either the pan-Turkic or the pan-Persian threats. The Kazakh protests even surfaced in Soviet-Iranian talks in Moscow in 1989 prompting Akbar Rafsanjani, the Iranian president, to clarify that Iran and the USSR ‘had agreed to not interfere in each other's affairs’.^90^

Nationalism and Islam-motivated tensions in the North Caucasus similarly surfaced in the 1970s and 1980s leading to concerns in the KGB. In one high-profile incident in 1971, villagers in a remote mountainous region in Dagestan occupied a mosque that had been repurposed for non-religious activities.^91^ After a stand-off that lasted for several weeks, the KGB intervened to quell the religious-national protest before it could spread. Similar problems were reported in Chechnya-Ingushetia. Throughout the Cold War, the KGB also remained concerned about the grievances against Soviet authorities among the North Caucasian Muslims such as Chechens and Ingushs who had been repressed under Stalin. The KGB monitored them closely and prevented them, including the supposedly loyal ‘registered clergy’, from being part of Hajj delegations for fear of them either defecting or spreading negative information about the USSR.^92^

By 1987, concern about Islam's resurgence entered the mainstream Soviet press, helped by *glasnost*. In an article on ‘Islam and Politics’ for *Literaturnaya Gazeta*, Igor Belyayev, a prominent journalist and frequent traveller in the Middle East, openly criticized the situation with Islam in Turkmenistan. Referring to ‘the dispiriting reality’ and ‘literally horrifying facts’ in Central Asia where local people went on pilgrimage to tombs of deserters who refused to fight in Afghanistan, Belyayev ‘gave every appearance of trying to make the flesh of his Russian readers creep’ according to the FCO. Belyayev linked this with direct involvement from abroad by the US and Iran. He referenced his conversation with Jalaleddin Farsi, an Iranian political leader, in Beirut in 1981 whom he claimed had threatened that ‘the Iranians would inspire a revolution in Central Asia’.^93^ This showed how many Soviets now saw an Iranian hand behind the rise of Islam in the Soviet Union. This concern had apparently crept into Soviet mainstream opinion from the KGB.

There were three main reasons that complicated the KGB's control over Islam in the USSR. One had to do with the difficulty of monitoring the practice of Islam in Central Asia and the Caucasus, especially in remote rural regions, due to the absence of ordination, a structured clergy and the need for consecrated grounds to perform rituals in Islam.^94^

Beyond this, the KGB felt that the USSR's Islamic diplomacy harmed the domestic situation with Islam. The visits of foreign Islamic authorities and the organization of Islamic conferences in the Soviet Union had led to increased religiousness among Soviet Muslims. The KGB noted how an Islamic conference in Uzbekistan in 1974 had ‘unexpected results’, forcing the authorities to conduct anti-religious measures more actively.^95^ Such incidents showed how, as with the Hajj, the Soviets had trouble managing the domestic and foreign connections in their policy toward Muslims.

A similar concern existed regarding the effect that contact with Iran could have on Soviet Muslims. In 1979, a rumour about the Iranian ambassador addressing a gathering of Muslims in Moscow spread among the diplomatic corps, making the Soviets uneasy. That same year, the Soviet ambassador to Iran speculated about bringing Iranian representation to Soviet Islamic conferences. However, even if such representation could be arranged, Iran's denunciation of the treatment of Soviet Muslims remained a concern.^96^

Another story had the Soviet Union in 1985 refusing to move the Iranian consulate from Leningrad to Dushanbe in Tajikistan. Moscow clearly did not want the Iranians to be directly in contact with the Tajiks, a Persian-speaking Iranian ethnic group.^97^ And, finally, testifying again to the influence Iran had on Azerbaijanis, reports indicated that Rafsanjani's address during Friday Prayers in Baku in 1989 led to widespread cheers as many attendees ‘wore black as a sign of mourning for Imam Khomeini’ who had recently died.^98^ Although Soviet-Iranian relations were by that point improving, such a display of emotion certainly reinforced the KGB's longstanding concern with the Islamic Revolution's negative influence on Soviet Muslims.

Third, the KGB remained distrustful of Soviet Islamic scholars. Although they saw the ‘unregistered clergy’ as the more problematic group, some in the KGB believed that SADUM was not active enough in curbing anti-Soviet activities, allowing unregistered scholars to prosper and being lax toward influences from abroad.^99^ Such suspicions remained traditionally associated with the Soviet institutions monitoring Islam which had to walk a tightrope between reporting on Islam and not appearing as if they had failed in their surveillance and control mission, especially given the poor knowledge of the situation with Islam by Moscow.

The Soviet authorities struggled to get the policy on Islam right during the last 20 years of the Soviet Union. The inter-relation of the domestic and foreign dimensions in the policy toward Muslims and the fact that Soviet policymakers in the Kremlin did not understand the situation in Muslim regions at the periphery of the Soviet Union made things more complicated. A tension remained: authorities in Moscow viewed Soviet Muslims and Islam with suspicion yet the Soviet Union pictured itself as a friend of Muslims worldwide and was keen to use Soviet Muslims to advance its foreign policy.

The USSR deployed Azerbaijanis, Tajiks, Uzbeks and other Soviet Muslims to Muslim countries where they made up a sizable proportion of Soviet operatives. Yet, although these agents had proven reliable over time, the Soviet authorities distrusted them. This diffuse suspicion showed in the absence of Muslims among KGB, MID and other institutions’ senior decision-makers in the field and in Moscow, and in the disparaging comments made by Slavs about their Muslim colleagues. In short, the KGB could not shake the idea that Soviet Muslims risked being instrumentalized by foreign powers, mainly Iran, against the Soviet state.

This tension between foreign and domestic policy imperatives surfaced when some of the Soviet initiatives to boost its influence in Muslim countries backfired at home. The KGB and SADUM sent pilgrims on Hajj, set-up Islamic conferences and invited Muslim officials to Soviet Muslim regions but this led to unforeseen burst of religiosity at home. This made the KGB anxious about a foreign hand and led it to strengthen anti-Islamic activities. It did not matter that religiosity was rarely directed against the Soviet authorities and, even when it was, it rarely led to direct action against them.

Conversely, Soviet Muslims had different experiences of interacting with the state. Some worked for it, others based their opposition to it in Islam. Among the former, although it is impossible to infer their motivations from their actions, some appeared to be sincere believers, ready to compromise for a chance to get an Islamic education abroad, go on Hajj or simply find an interesting job. Others faithfully and wholeheartedly served the state, joining the MID or KGB to become operatives in Muslim countries and, on returning home, agents in running mosques and joining Islamic institutions in Muslim regions. The Mitrokhin Archives recount the story of two generations of Islamic scholars in Tajikistan and Uzbekistan who were KGB agents. This may be indicative of a pattern, especially knowing that Islamic education often happened in familial circles in Central Asia.^100^

Ultimately, it is clear that KGB penetration of SADUM and its equivalents was considerable, creating a fragile balance between Islam and communism. That status quo began unravelling in the late 1980s as Soviet power weakened. A new generation of Islamists would then see the older generation of Islamic scholars as having compromised too much during Soviet times, especially because of its dealings with the KGB, and foster the fragmentation of the religious field in post-Soviet Muslim regions. Showing the relevance of the Soviet legacies to post-Soviet politics, different generations of Islamic scholars would then align with either opposition forces or neo-communist and pro-Russian elites in the conflicts in Chechnya, Tajikistan and Uzbekistan.

## Notes

1 In the late 1970s, out of the 50 Soviet diplomats in Iran, 23 were ‘clean’, in KGB parlance, 15 were KGB agents, and 12 were agents of military intelligence (GRU). Among the 23 ‘clean’ diplomats, 20 were informers for the KGB. Vladimir Kuzichkin and Galina Kuzichkina, *KGB. Mify i real’nost’. Vospoeminaniya sovetskogo razvedchika i ego zheny* (Moscow: Eksmo, 2023), 147–68.

2 Christopher Andrew and Vasili Mitrokhin, *The World Was Going Our Way: The KGB and the Battle for the Third World* (New York: Basic Books, 2006).

3 Dmitry Asinovskiy, ‘“A Priest Does Not Consider the Toppling of the Shah as an Option” The KGB and the Revolution in Iran,’ *Iranian Studies* 1 (2022), 3.

4 See: Alexandre Bennigsen and Chantal Lemercier-Quelquejay, *Le Soufi et le commissaire: Les confréries musulmanes en URSS* (Paris: Seuil, 1986); Artemy M. Kalinovsky, ‘Encouraging Resistance: Paul Henze, the Bennigsen School, and the Crisis of Détente,’ in Michael Kemper and Artemy M. Kalinovsky (eds) *Reassessing Orientalism*, *Interlocking Orientologies during the Cold War* (London: Routledge, 2015), 211–32.

5 Eren Tasar, ‘Mantra: A Review Essay on Islam in Soviet Central Asia,’ *Journal of the Economic and Social History of the Orient* 63, 3 (2020), 395, 406. Also, Botakoz Kassymbekova and Aminat Chokobaeva, ‘On Writing Soviet History of Central Asia: Frameworks, Challenges, Prospects,’ *Central Asian Survey*, 40, 4 (2021), 483–503; Devin Deweese, ‘Islam and the Legacy of Sovietology: A Review Essay on Yaacov Ro’i’s Islam in the Soviet Union,’ *Journal of Islamic Studies*, 13, 3 (2002), 298–330; Yaacov Ro’i, *Islam in the Soviet Union, From the Second World War to Gorbachev* (London: Hurst, 2000); Paolo Sartori, *A Soviet Sultanate: Islam in Socialist Uzbekistan (1943–1991)* (Vienna: Austrian Academy of Sciences Press, 2024).

6 Tasar, ‘Mantra’, 411.

7 Paolo Sartori, ‘Why Soviet Islam Matters,’ in Paolo Sartori (ed.) *Soviet and Post-Soviet Islam: State of the Field and New Directions* (Wien: Verlag der österreichischen Akademie der Wissenschaften, 2023), 21.

8 Sartori, ‘Why Soviet Islam Matters,’ 15.

9 Sartori, ‘Why Soviet Islam Matters,’ 6; Also, Sartori, *Soviet Sultanate*, 23.

10 Sartori, ‘Why Soviet Islam Matters,’ 6.

11 Eren Tasar, *Soviet and Muslim, The Institutionalization of Islam in Central Asia* (London: Oxford University Press, 2017), 107–08, 125, 183–87, 345–58. The term ‘clergy’ makes little sense for Sunni Islam. This article uses instead the term ‘Islamic scholars’ unless it quotes Soviet sources.

12 Sartori, *Soviet Sultanate*, 4. Also, Bakhtiyar M. Babadzhanov, Ashirbek K. Muminov, and Martha Brill Olcott, “Mukhammadzhan Khindustani (1892–1989) i religioznaya sreda ego epokhi,” *Vostok* (*Oriens*), 5 (2004), 43–59.

13 Sartori, *Soviet Sultanate*, 32–33, 273.

14 Tasar, *Soviet and Muslim*, 4.

15 Fred Halliday, ‘“Islam” and Soviet Foreign Policy,’ *Journal of Communist Studies*, 3, 1 (1987), 217–33; Masha Kirasirova, ‘“Sons of Muslims” in Moscow: Soviet Central Asian Mediators to the Foreign East, 1955–1962,’ *Ab Imperio*, 4 (2011), 106–32; Masha Kirasirova, *The Eastern International, Arabs, Central Asians, and Jews in the Soviet Union's Anticolonial Empire* (London: Oxford University Press, 2024); Tasar, *Soviet and Muslim*, 243.

16 National Archives, London (NA), Foreign and Commonwealth Office (FCO) (now Foreign, Commonwealth and Development Office) 93/2292, 1980, item (i.) 23; NA FCO93/2622, 1980, i.1; NA FCO93/3595, 1983, i.74; NA FCO93/5866, 1989, i.37a.

17 Sartori, *Soviet Sultanate*, 268. Also, Artemy M. Kalinovsky, *Laboratory of Socialist Development: Cold War Politics and Decolonization in Soviet Tajikistan* (Ithaca: Cornell University Press, 2018), Kindle; Tasar, *Soviet and Muslim*, 298–364.

18 Vassily Klimentov, *A Slow Reckoning: The USSR, the Afghan Communists, and Islam* (Ithaca: Northern Illinois University Press, 2024), 180–226; Yaacov Ro’i, *The Bleeding Wound, The Soviet War in Afghanistan and the Collapse of the Soviet System* (Redwood City: Stanford University Press, 2022), 251–78; Sartori, *Soviet Sultanate*, 33.

19 Sartori, *Soviet Sultanate*, 268; Tasar, *Soviet and Muslim*, 298–364.

20 Adeeb Khalid, *Central Asia: A New History from the Imperial Conquests to the Present* (Princeton: Princeton University Press, 2021), 305–54, 377–90; Adeeb Khalid, ‘Islam in Central Asia 30 years after independence: debates, controversies and the critique of a critique,’ *Central Asian Survey*, 40, 4 (2021), 539–54.

21 Stéphane Dudoignon, ‘Gnosis as Dissent? In Soviet and Present-Day Tajikistan,’ *Journal of Central Asian History*, 1, 2 (2022), 273–308.

22 Anatoly Chernyaev, *The Diary of Anatoly Chernyaev* (Washington, DC: National Security Archive, 2003), 14 July 1984, October 1984.

23 Andrei Gromyko, *Pamyatnoe, Kniga vtoraya* (Moscow: Politizdat, 1990), 286–87.

24 Timothy Nunan, *Humanitarian Invasion, Global Development in Cold War Afghanistan* (London: Cambridge University Press, 2016), 99–100.

25 Klimentov, *A Slow Reckoning*, 67–70, 209.

26 Chernyaev, *Diary, 27 August 1985.*

27 Churchill Archives Centre, University of Cambridge (CAC), MITN1/4, Volume (V.) 4, 46–47.

28 CAC, MITN1/5, V.5, 8, 20. Also, Vyacheslav Akhmadullin, ‘The 1966–1967 Hajj of Soviet Muslims: Analysing Archival Documents,’ *Oriental Studies*, 16, 3 (2023), 504–15; Eileen Kane, *Russian Hajj, Empire and the Pilgrimage to Mecca* (Ithaca: Cornell University Press, 2020).

29 Vyacheslav Akhmadullin, ‘The Hajj of Muslims of the North Caucasus (1944–1991),’ *History, Archaeology and Ethnography of the Caucasus*, 19, 3 (2023), 732–42; Tasar, *Soviet and Muslim*, 245–46, 266–68.

30 CAC, MITN1/5, V.5, 16–17. Also, Sartori, *Soviet Sultanate*, 293.

31 CAC, MITN1/5, V.5, 16–17; CAC, MITN1/4, V.5, 121; CAC, MITN1/3, V.3, 111. In this, the KGB’s approach to Soviet Muslims was no different from its approach to other religious groups who had co-religionaries abroad such as Buddhists.

32 A partial list of KGB resident spies per country during the Cold War is available on: https://shieldandsword.mozohin.ru/kgb5491/resident.htm.

33 NA, FCO93/1315, 1977, i.4–5. Also, NA, FCO93/2257, 1979, i.5–8.

34 A Soviet Muslim led a delegation to Jordan in 1982: NA, FCO93/3080, 1982, i.3. The preference given to Slavs compared to Soviet Muslims regarding most attractive MID postings in the Middle East has been confirmed in an interview by a former Uzbek interpreter in South Yemen. Robert O. Freedman, Communication with author, December 2024.

35 Ro’i, *The Bleeding Wound*, 9–25.

36 NA, FCO93/3941, 1984, i.6,11; NA, FCO93/3976, 1984, i.5, 9.

37 NA, FCO93/1695, 1978, i.37.

38 For an FCO visit to the Oriental Studies Institute, see: NA, FCO93/1695, 1978, i.18. Also, Vadim Kirpichenko, *Razvedka: Litsa i Lichnosti* (Moscow: Mezhdunarodnye Otnosheniya, 2017), 222, 360–61. Kirpichenko discusses the people who handled the Middle East in the KGB. He notes how many KGB agents passed through the Oriental Studies Institute.

39 CAC MITN1/2, V.2, 43. Also, CAC MITN1/2, V.2, 63; CAC MITN1/2, V.2, 44, 50.

40 CAC MITN1/2, V.2, 21; CAC MITN1/2, V.2, 23.

41 NA, FCO8/2729, 1976, i.1–5, 13; Kuzichkin and Kuzichkina, *KGB*, 298–320.

42 CAC MITN1/2, V.2, 38; Asinovskiy, ‘“A Priest Does Not Consider’, 1–23; Dmitry Asinovskiy, ‘Rostislav Ulianovskii, the Tudeh Party of Iran and Soviet Attempts to set Iran on a Non-Capitalist Path of Development (1979–83),’ *Cold War History*, 24, 1 (2024), 45–65; Jeremy Friedman, *Ripe for Revolution: Building Socialism in the Third World* (Cambridge: Harvard University Press, 2022); Timothy Nunan, ‘“Doomed to Good Relations”: The USSR, the Islamic Republic of Iran, and Anti-Imperialism in the 1980s,’ *Journal of Cold War* Studies, 24, 1 (2022), 39–77; Leonid Shebarshin, *Ruka Moskvy, Zapiski nachal’nika vneshnei razvedki* (Moscow: Algoritm, 2017), 183.

43 CAC MITN1/2, V.2, 44; CAC MITN1/2, V.2, 40.

44 NA, FCO93/2622, 1980, i.1.

45 Russian State Archive of Contemporary History (RGANI), 1980, file (f.) 89, opis’ (o.) 34, delo (d.) 3, list(y) (ll.) 1–8; CAC MITN1/2, V.2, 22, 54, 97B; CAC MITN1/4, V.4, 61–63; CAC MITN1/2, V.2, 57; Roland Dannreuther, *The Soviet Union and the PLO* (London: Macmillan, 1998).

46 CAC MITN1/3, V.3, 2–4. See also: https://shieldandsword.mozohin.ru/kgb5491/resident.htm; Kuzichkin and Kuzichkina, *KGB*, 127–46.

47 CAC MITN1/2, V.2, 37, 82.

48 NA, FCO8/3580, 1980, i. 25, i. 30, i.36; NA, FCO8/4574, 1982, i.8; RGANI, 1985, f 89, o.15, d.14, ll.1–5.

49 Kuzichkin and Kuzichkina, *KGB*, 339–84.

50 Kuzichkin and Kuzichkina, *KGB*, 127–46, 321–30.

51 Kuzichkin and Kuzichkina, *KGB*, 147–68, 231–76.

52 RGANI, 1980, f.89, o.32, d.33, ll.1–4; RGANI, 1980, f.89, o.43, d.2, ll.1–2; RGANI, 1984, f.89, o.15, d.12, ll.1–5; RGANI, 1985, f.89, o.15, d.14, ll.1–5; RGANI, 1985, f.89, o.15, d.22, ll.1–5.

53 Kuzichkin and Kuzichkina, *KGB*, 384–405, 406–30; RGANI, 1986, f.89, o.13, d.7, ll.1–12.

54 Kuzichkin generally describes his Muslim colleagues as lazy, corrupt, and incompetent.

55 NA, FCO8/4574, 1982, i.21–22. Also, Asinovskiy, ‘Rostislav Ulianovskii,’ 45–65.

56 NA, FCO8/4574, 1982, i.57–58, 60, 62. Also, NA, FCO8/3371, 1979, i.57–58, 60, 62; Also, NA, FCO8/3372, 1979, i.58.

57 CAC MITN1/3, V.3, 18, 23, 26.

58 CAC MITN1/3, V.3, 1–8.

59 CAC MITN1/3, V.3, 23–25; CAC MITN1/3, V.3, 20–21, 34, 65–66.

60 CAC MITN1/3, V.3, 30–32, 43; Klimentov, *Slow Reckoning*, 170–78.

61 CAC MITN1/3, V.3, 102D, 102G; CAC MITN1/3, V.3, 110.

62 CAC MITN1/4, V.4, 54, 61–63.

63 CAC MITN1/4, V.4, 8–9.

64 Olivier Roy, Interview by author, December 2019. Olivier Roy is a prominent expert on Afghanistan who conducted field research among the Mujahideen in the 1980s.

65 Sartori, *Soviet Sultanate*, 323.

66 Klimentov, *Slow Reckoning*, 91–96; Ro’i, *Bleeding Wound*, 251–78.

67 Sartori, *Soviet Sultanate*, 294.

68 RGANI, 1985, f.89, o.15, d.14, ll.1–5.

69 CAC MITN1/5, V.5, 20.

70 Vladimir Plastun, Interview by author, E-mail, May 2019/April 2020. Plastun was a senior adviser with the Soviet military in Afghanistan.

71 RGANI, 1988, f. 117, o. 1, d. 17, ll. 2–13 quoted in Klimentov, *Slow Reckoning*, 70.

72 ‘Afganskaya vakhta Tabeeva,’ *Business-Gazeta.ru*, 3 March 2013. https://www.business-gazeta.ru/article/76456. Also: Klimentov, *Slow Reckoning*, 56; Nina Shishkina and Irina Yakovleva, *Fikryat Tabeev. Sud’be blagodarya i vopreki* (Kazan: TKI, 2017), 240–93.

73 KGB Senior Lieutenant, Interview by author, Moscow, Feb 2019. The Senior Lieutenant worked on Iran in the KGB in Moscow.

74 Vladimir Kryuchkov, *Lichnoe Delo*, *Chast’ Pervaya* (Moscow: Olimp, 1996), 200.

75 See: Vitaly Naumkin, *Radical Islam in Central Asia: Between Pen and Rifle* (Lanham, MD: Rowman & Littlefield, 2005); Martha Brill Olcott, *Roots of Radical Islam in Central Asia* (Washington, DC: Carnegie, 2007); Vassily Klimentov, ‘The Tajik Civil War and Russia’s Islamist Moment,’ *Central Asian* Survey, 42, 2 (2023), 341–58; Sartori, *Soviet Sultanate*, 27, 280, 313; Emil Souleimanov, *An Endless War: The Russian-Chechen Conflict in Perspective* (Bern: Peter Lang, 2007).

76 NA, FCO8/3372, 1979, i.53; NA, FCO8/3372, 1979, i.63, 83, 85.

77 NA, FCO8/4715, 1986, i.1. Also, NA, FCO8/5893, 1985, i.22; NA, FCO8/6262, 1987, i.13.

78 NA, FCO8/7583, 1989, i.16.

79 NA, FCO8/6276, 1990, i.6.

80 CAC MITN1/5, V.5, 10–11.

81 Akhmadullin, ‘The 1966–1967 Hajj’.

82 CAC MITN1/5, V.5, 20; Tasar, *Soviet and Muslim*, 268–72.

83 Dudoignon, ‘Gnosis as Dissent?’, 19. Also, Sartori, *Soviet Sultanate*, 307.

84 CAC MITN1/5, V.5, 18–19, 49

85 GARF, 26 Oct–24 Dec 1981, f.R6991, o.6, d.2206, l.11.

86 Kirpichenko, *Razvedka*, 161.

87 CAC MITN1/5, V.5, 11; CAC MITN1/5, V.5, 8–9. Also, Dudoignon, ‘Gnosis as Dissent?’, 19; Sartori, *Soviet Sultanate*, 306–07.

88 Davlat Khudonazarov, Interview by author, Moscow, Feb 2019. Khudonazarov is a Tajik film director who was the candidate for the Islamist-democratic block at the Tajik presidential election in 1991.

89 James Critchlow, *Nationalism in Uzbekistan: A Soviet Republic’s Road to Sovereignty* (Boulder: Westview Press, 1991); Riccardo Mario Cucciolla, ‘Sharaf Rashidov and the International Dimensions of Soviet Uzbekistan,’ *Central Asian Survey*, 39, 2 (2020), 185–201; Adeeb Khalid, *Central Asia: A New History from the Imperial Conquests to the Present* (Princeton: Princeton University Press, 2021), 377–90.

90 NA, FCO8/6262, 1986–87, i.14–16. Also, CAC MITN1/5, V.5, 45.

91 CAC MITN1/5, V.5, 25–29, 30–40.

92 Akhmadullin, ‘The Hajj of Muslims’.

93 NA, FCO8/6630, 1987, i.10a.

94 CAC MITN1/5, V.5, 21; Tasar, *Soviet and Muslim*, 51.

95 CAC MITN1/5, V.5, 10, 17.

96 NA, FCO8/3372, 1979, i.68, 71, 75, i.84A.

97 NA, FCO8/5893, 1985, i.10.

98 NA, FCO8/7413, 1989, i.17b.

99 CAC MITN1/5, V.5, 42–44, 51.

100 CAC MITN1/5, V.5, 42-44; Sartori, *Soviet Sultanate*, 286–87.

